# Gut microbial dysbiosis activates the classical complement pathway in a short-term morphine treatment model

**DOI:** 10.1080/29933935.2025.2527628

**Published:** 2025-07-13

**Authors:** Nicolas Vitari, Richa Jalodia, Junyi Tao, Udhghatri Kolli, Salma Singh, Lily V. Rodriguez, Umakant Sharma, Sabita Roy

**Affiliations:** a Department of Microbiology and Immunology, University of Miami Miller School of Medicine, Miami, USA; b Department of Surgery, University of Miami Miller School of Medicine, Miami, USA

**Keywords:** IgM, IgG3, classical complement pathway, morphine, microbiome

## Abstract

Antibodies play an essential role in preserving intestinal homeostasis in healthy and dysbiotic states. Recent studies demonstrate that a microbiome-dependent intestine-specific complement system maintains intestinal homeostasis. Morphine induces microbial dysbiosis within hours of administration characterized by the expansion of pathogenic bacteria with a concurrent decrease in commensal bacteria. A murine model of short-term morphine treatment was used to provide insights into the early immune processes during microbial dysbiosis. Within 24 h, morphine treatment upregulates the expression of classical complement pathway genes in intestinal tissue, with a concurrent increase in the complement proteins C1q and C3 in the ileal luminal content. Importantly, a parallel increase in the concentration of complement-activating antibodies IgM and IgG is observed in the ileal luminal content at 24 h. The increased concentration of complement proteins and antibodies is dependent on the microbiome, as microbial depletion prior to morphine treatment abolishes this increase. Finally, intestinal infiltration and activation of neutrophils is observed concurrent with microbial dysbiosis. This study demonstrates rapid microbiome-dependent intestinal recruitment of complement machinery during microbial dysbiosis. Together, these data confirm the relationship between intestinal complement and the microbiome and show that the classical complement system is activated to protect the host during microbial dysbiosis.

## Introduction

Trillions of bacteria, viruses, and fungi comprise the intestinal microbiome. Maintaining the composition of the microbiome is essential for human health. During chronic disease, the composition of the microbiome becomes disrupted.^1^ Multiple immune mechanisms cooperate in response to microbial dysbiosis, particularly secretory immunoglobulins.^2^ Recently, an intestinal complement system independent of circulation was described to selectively remove intestinal pathogens while sparing commensals.^3^

The classical complement pathway is activated when either IgM or IgG binds to an antigen.^4^ Antibody-bound microbes may be directly killed by myeloid cells or activate the complement cascade.^5^ Due to its pentameric structure, IgM fixes complement extremely efficiently.^6^ Though less efficient than IgM, even a low concentration of mouse IgG3 activates the complement cascade.^7^ Upon antibody recognition of an antigen, an immune complex (IC) forms which recruits complement proteins for clearance.^8^ Complement component 1q (C1q) combines with C1r and C1s to form the C1 complex.^4^ The C1 complex initiates the complement cascade by splitting C4 to finally cleave C3. The alternative and lectin pathways are initiated via different mechanisms and converge with the classical pathway at C3, making the C1 complex distinctive of the classical pathway and C3 a key contributor to complement activation.^4^ C1q and C3 play important, non-redundant roles during homeostasis and intestinal inflammation.^3,9–11^ The complement fragment C3b is an opsonin that deposits on the surface of microbes which results in either direct killing by neutrophils or macrophages, or activation of the membrane attack complex (MAC).^12^ C3 also plays important neural functions that are dependent on the microbiome.^13^ While IgA dominates at homeostatic conditions, intestinal IgM and IgG are present at low concentrations, if at all.^14–16^ However, microbial dysbiosis often leads to an increased intestinal antibody response.^17–19^ Additionally, complement-deficiency in mice results in intestinal complications, and complement genes are enriched in inflammatory bowel diseases.^19–22^ Together, it is plausible that IgM and IgG could be recruited to the intestine to activate the classical complement system in direct response to microbial dysbiosis.

Opioids are the gold standard for moderate to severe pain management.^23^ However, opioids such as morphine cause many negative intestinal side effects including gut barrier disruption and microbial dysbiosis^24,25^ within 24 h of administration. Microbial dysbiosis following morphine treatment is characterized by expansion of potentially pathogenic gram-positive bacteria and a decrease in commensal bacteria.^26^ Rapid production of IgM and IgG3 can be induced by toll-like receptor (TLR) stimulation of B cells.^27,28^ Notably, TLR signaling is involved in morphine-induced microbial dysbiosis, which could potentially induce an increase in IgM and IgG.^29–31^ Morphine-induced microbial dysbiosis is accompanied by activation of intestinal B cells and infiltration of myeloid cells to the intestinal lamina propria.^29,32^ Myeloid cells, particularly neutrophils, are essential players in complement-mediated clearance of intestinal pathogens.^3^ Therefore, morphine-induced microbial dysbiosis may result in rapid recruitment of intestinal complement system components to limit the expansion of pathogenic bacteria.

Here, we report that morphine induces upregulation of classical complement-related genes in intestinal tissues with a corresponding increase in the concentration of C1q and C3 in the ileal luminal content. Next, we report an increase in the concentration IgM, IgG3, and ICs in the ileal luminal content following morphine treatment. We then demonstrate that the increase in IgM, IgG, and complement proteins is dependent on the microbiome. Further, we report that intestine-infiltrating neutrophils increase CD11b expression at 24 h of morphine treatment. Together, these data illustrate a novel role for the classical complement system during the early stages of microbial dysbiosis.

## Materials and methods

### RNA-seq analysis

Differentially expressed genes (DEGs) were retrieved from our previously published data set.^26^ The clusterProfiler^33^ R package was used to test the statistical enrichment of differential expression genes in Kyoto Encyclopedia of Genes and Genomes (KEGG)^34^ pathways. KEGG terms with corrected *p* value less than 0.05 were considered significantly enriched. To visualize the expression of complement-related genes, DEGs were log-transformed, Z-scores were calculated, and a heat map of Z-scores was generated using Prism.

### Animals

Wild-type male or female C57BL/6 and BALB/c mice were purchased from Jackson Laboratories (Accession #: 000664 and 000651). Prior to experimentation, mice were acclimated for at least 1 week. Mice were held in specific-pathogen-free conditions with sterile water and food provided ad libitum. All animal experiments were approved by the University of Miami Institutional Animal Care and Use Committee (IACUC). The experiments were performed in compliance with the institutional laws and guidelines.

### Morphine treatment

Twelve- to sixteen-week-old mice were lightly anesthetized with isoflurane (Pivetal®) and subcutaneously implanted with a continuous release 25 mg morphine pellet or placebo pellet for 24 or 48 h. The slow-release 25 mg morphine pellet emulates the plasma concentration of opioid use in the clinic and is a well-established murine model for morphine dependence.^35,36^ Morphine and placebo pellets were obtained from National Institute on Drug Abuse. All efforts were made to minimize suffering during and after surgery.

### Microbiome depletion

The intestinal microbiome was depleted with a previously described antibiotic cocktail.^29–31^ Briefly, mice were orally fed 0.2 ml of antibiotic cocktail or sterile water once per day for 7 days prior to implantation of 25 mg slow-release morphine pellet. The antibiotic cocktail consisted of 10 mg/mL bacitracin, 10 mg/mL metronidazole, 40 mg/mL neomycin sulfate, 4 mg/mL vancomycin, and 24 µg/mL pimaricin dissolved in water.

### Intestinal luminal content processing and ELISA

Proteins in the intestinal luminal content were quantified via ELISA as previously described with minimal modifications.^3,29,37^ Briefly, intestinal luminal contents were squeezed out using sterile forceps, weighed, and frozen at −80℃. Frozen luminal contents were diluted to 100 mg/ml in sterile PBS with 1% protease inhibitor cocktail (Invitrogen; catalog no. 78429) and vortexed for 20 min at 4℃. After centrifugation at 7000 × g for 15 min, supernatant was collected and diluted prior to ELISA analysis. Dilutions for luminal content samples were determined empirically. Total IgM and IgG3 were measured using ELISA kits from Invitrogen (Catalog nos. 88–50470–22, 88–50440–22, 88–50430–88) following the manufacturer’s protocol. C1q and C3 were measured using ELISA kits from Abcam (Catalog nos. ab291069, ab157711) following the manufacturer’s protocol. ICs were measured using an ELISA kit from Biomatik (Catalog no. EKC36559-96T) following the manufacturer’s protocol.

### Immune cell flow cytometry

Intestinal immune cells were detected via flow cytometry as previously described.^32^ Briefly, immune cells from the ileum lamina propria were isolated using the Lamina Propria Dissociation Kit (Miltenyi Biotec; catalog no. 130–097–410) according to the manufacturer’s recommendations. Following isolation, immune cells were washed with PBS and resuspended in PBS + 1%BSA and stored at 4°C before proceeding with flow cytometry staining. For flow cytometry staining, ~1 × 10^6^ cells were placed in a 96-well plate with Fc block (BD biosciences) and viability dye for 15 min at 4°C. Cells were then stained with antibody mixture for surface staining at 4°C for 30 min. Cells were washed after staining with staining buffer and fixed with fixation buffer (Biolegend). Cells were analyzed on BD LSR II (BD biosciences) and flow cytometry data was analyzed using FlowJo software (version 10.8.0; Tree star, Ashland, OR).

### Statistical analysis

Prism 10.0.1 was used to perform the statistical analysis of ELISA and flow cytometry data. Details on the analyses done for each plot can be found in the corresponding figure legend. Symbols represent individual mice. Mean and standard deviation are shown. Stars denote the following *p*-values: *p < 0.05 **p < 0.01 ***p < 0.001 ****p < 0.0001.

## Results and discussion

### Morphine upregulates the classical complement system in the intestine

**Figure 1.**
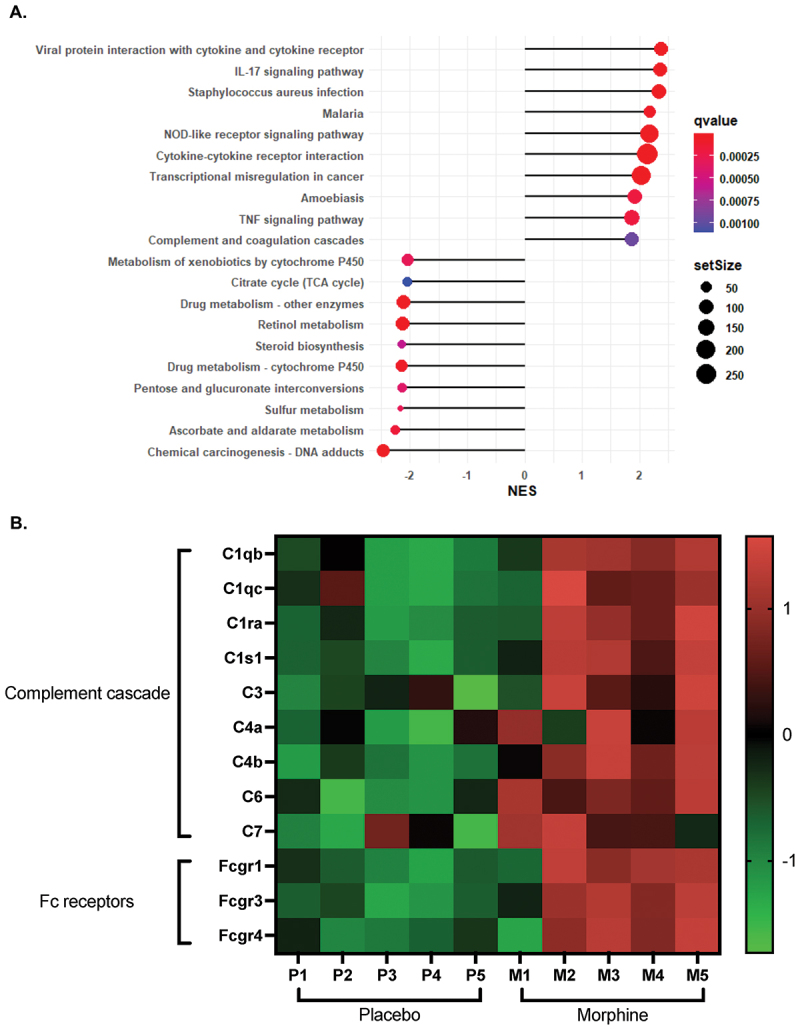
Increased intestinal expression of classical complement genes during morphine treatment.

Morphine treatment causes rapid changes in the intestinal microbiome, accompanied by both transcriptomic and metabolomic alterations.^26^ Using our previously published data set,^26^ we show that the complement pathway was one the most upregulated pathways in small intestinal tissue during morphine treatment (Figure 1A). Importantly, the complement cascade can be initiated by three independent mechanisms – the classical, mannose binding lectin (MBL), or alternative pathways – which are each driven by distinct genes.^4^ To further investigate how complement-related genes are altered, and which complement pathways are upregulated, we generated a heatmap of DEGs related to the complement cascade. Many genes involved in the complement cascade were upregulated, particularly components of the C1 complex (Figure 1B). Expression of important genes in the lectin (*Masp1/2*, *Mbl2*) and alternative (*Cfd*, *Cfp*) pathways were not significantly changed with morphine treatment (Table S1).^26^ Further, expression of *Cfb* was significantly reduced, while expression of the inhibitor *Cfh* was significantly increased, suggesting that activation of the complement cascade proceeds independently of the alternative pathway (Table S1).^26^ C8 and C9 are required for MAC-dependent lysis of bacteria.^12^ Consistent with previous reports, the expression of intestinal C8 and C9 were unchanged during morphine treatment, suggesting that the MAC fails to form (Table S1).^3,26,38^ Fc receptor gene expression was increased, which may indicate increased recognition of antibody-bound microbes or myeloid cell infiltration (Figure 1B). Together, these data suggest that morphine upregulates the intestinal complement pathway and implicates classical activation.

(A). Lollipop plot of the ten most up- and down-regulated pathways based on Normalized Enrichment Score (NES) using GSEA analysis. Data isolated from a previously published RNAseq data set of murine small intestinal tissue treated with a 25 mg morphine or placebo pellet for 24 h [26] (B). Heat map of differentially expressed complement-related genes. All genes have a *p*-value <0.05 (*n* = 5) and are significantly upregulated in the morphine-treated samples.

**Figure 2.**
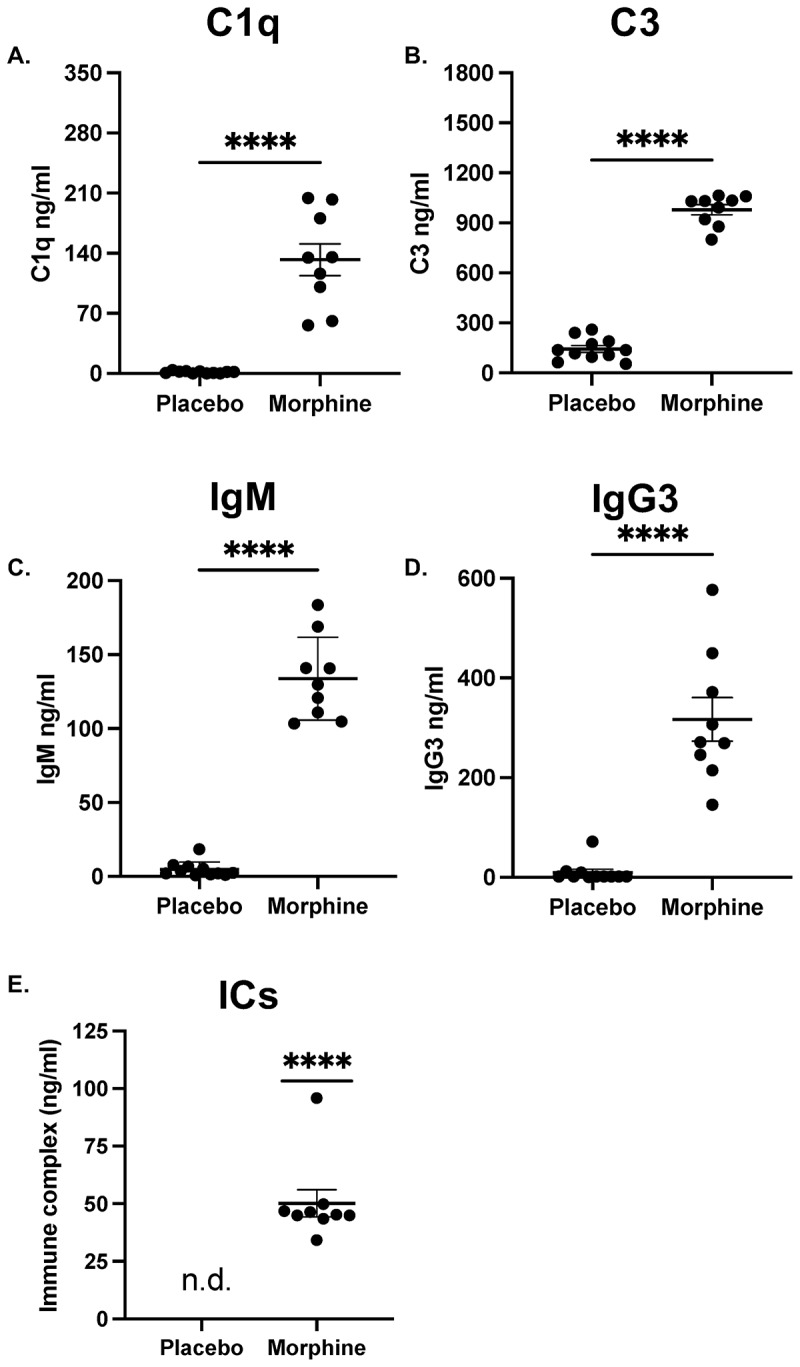
The concentrations of C1q, C3, IgM, IgG3, and ICs increase in the ileal luminal content during morphine treatment.

To validate that the classical complement proteins are present in the intestines, C1q and C3 were measured in the ileal luminal content of morphine-treated mice.^3^ Indeed, there was a significant increase in the concentration of both C1q and C3 during morphine treatment (Figure 2A,B). These data confirm that there is an increase in intestinal complement and further implies activation of the classical pathway.

Concentration of (A). C1q, (B). C3, (C). IgM, (D). IgG3, and (E). Immune complexes (ICs) determined by ELISA from the ileal luminal content of mice 24 h after implantation with 25 mg morphine or placebo pellet (*n* = 9–11). Symbols represent individual mice. Mean and standard error of the mean (SEM) are shown. Data points are pooled from three independent experiments. *p < 0.05 **p < 0.01 ***p < 0.001 ****p < 0.0001 using Mann Whitney U test (A-D) or One sample Wilcoxon test (E). n.d. = not detected.

### The concentration of IgM, IgG3, and ICs increase in the ileal luminal content during morphine treatment

The murine classical complement system is initiated by antibodies. To further investigate the potential for classical complement activation, we measured the concentration of IgM and IgG in the ileal luminal content following 24 h of morphine treatment. Since IgM is the most potent activator of complement, we first measured the concentration of intestinal IgM. The concentration of IgM was significantly increased in the ileal luminal content following morphine treatment (Figure 2C). Due to its oligomerization ability, IgG3 activates complement the most potently of all murine IgGs.^7^ The concentration of IgG3 was greatly increased in the ileal luminal content of morphine-treated mice compared to placebo-treated controls (Figure 2D). Morphine-induced activation of the classical complement system is not dependent on strain or sex, as the concentration of both IgM and C1q was elevated in the ileal luminal content of BALB/c and female B6 mice (Fig S1A-D). Together, these data illustrate that morphine causes an increased concentration of IgM and IgG3 which further implicates activation of the classical complement system.

Antibodies must bind to antigen, forming ICs, to efficiently activate complement.^39^ Upon IC formation, IgM and IgG undergo a conformational change which reveals a binding site for complement proteins.^40^ To validate that the intestinal antibodies present during morphine treatment are capable of activating complement, we measured the concentration of ICs. ICs were present in the ileal luminal content of morphine-treated animals and absent from placebo-treated controls, further supporting intestinal activation of the classical pathway during morphine treatment (Figure 2E).

Opioid-induced microbial dysbiosis persists for multiple days.^41^ However, the alterations to intestinal IgA are limited to the ileum despite morphine causing microbial dysbiosis throughout the intestine.^29,41^ Therefore, we hypothesized that the increased concentration of intestinal antibodies may be detectable at later time points. IgM and IgG3 remained elevated in the ileal luminal content at 48 h of morphine treatment (Fig S2A and B). We further investigated if the concentrations of IgM and IgG3 are increased in the large intestinal luminal content. Interestingly, IgM and IgG3 were elevated in the large intestinal luminal content at 24 h of morphine treatment (Fig S3A and B). However, only the concentration of IgM was increased at 48 h in the large intestinal luminal content (Fig S3C and D). The concentration of all antibodies at 48 h in the ileal luminal content and in the large intestinal luminal content at both time points were lower than in the ileal luminal content at 24 h (Figure 2C,D). Morphine induces gut barrier disruption in the ileum, but not in the large intestine, which may contribute to the concentration difference between small and large intestine.^25^ Considering the comparatively low number of intestinal IgM- and IgG-secreting cells in mice,^42–44^ the increased concentration of antibodies could originate from circulation and leak into the lumen due to barrier disruption. Together, these data demonstrate that the concentration of intestinal IgM and IgG3 are increased throughout the intestine and may play a distinct role from IgA during morphine-induced microbial dysbiosis.

### The microbiome is required for the morphine-induced increased concentration of IgM, IgG3, and complement

**Figure 3.**
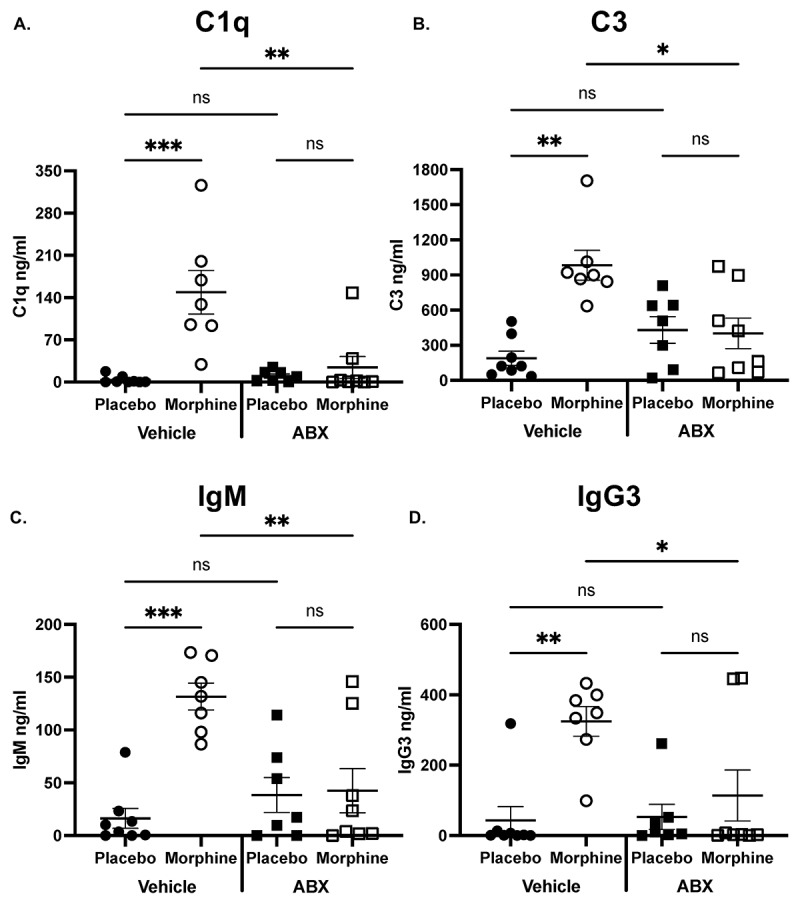
The microbiome is required for morphine-induced increased intestinal IgM, IgG, and complement.

Complement is essential to maintaining intestinal homeostasis, and the microbiome was recently discovered to directly modulate the concentration of intestinal C3.^3^ Since morphine causes microbial dysbiosis, we investigated whether an intact microbiome is required for the morphine-induced increase in concentration of C1q and C3. Mice were treated with an antibiotic cocktail that has been previously established to deplete the microbiome.^31^ After treatment with an antibiotic cocktail or water, mice were implanted with a 25 mg morphine or placebo pellet and sacrificed after 24 h. Microbiome depletion abrogated the increased concentration of C1q and C3 in the ileal luminal content (Figure 3A,B). Additionally, microbiome depletion abolished the increased concentration of IgM and IgG3 (Figure 3C,D). These data confirm there is a relationship between intestinal recruitment of classical complement components and the intestinal microbiome. Considering our recent work on IgA-bacterial dynamics during morphine-induced microbial dysbiosis,^29^ the data presented here support the assertion that IgM, IgG, and IgA act in concert to maintain microbial homeostasis.^2,15,45^ Further, this study establishes morphine treatment as a valuable mouse model for elucidating the synergistic effects of intestinal antibodies and complement on the microbiome.

Concentration of (A). C1q, (B). C3, (C). IgM, and (D). IgG3 determined by ELISA from the ileal luminal content of microbiome-depleted or vehicle-treated mice following 24 h of continuous morphine treatment (*n* = 7–8). Symbols represent individual mice. Mean and SEM are shown. Data points are pooled from three independent experiments. *p < 0.05 **p < 0.01 ***p < 0.001 ****p < 0.0001 using Two-way analysis of variance with Tukey correction (A-D).

### Morphine causes increased CD11b expression on intestine-infiltrating neutrophils

**Figure 4.**
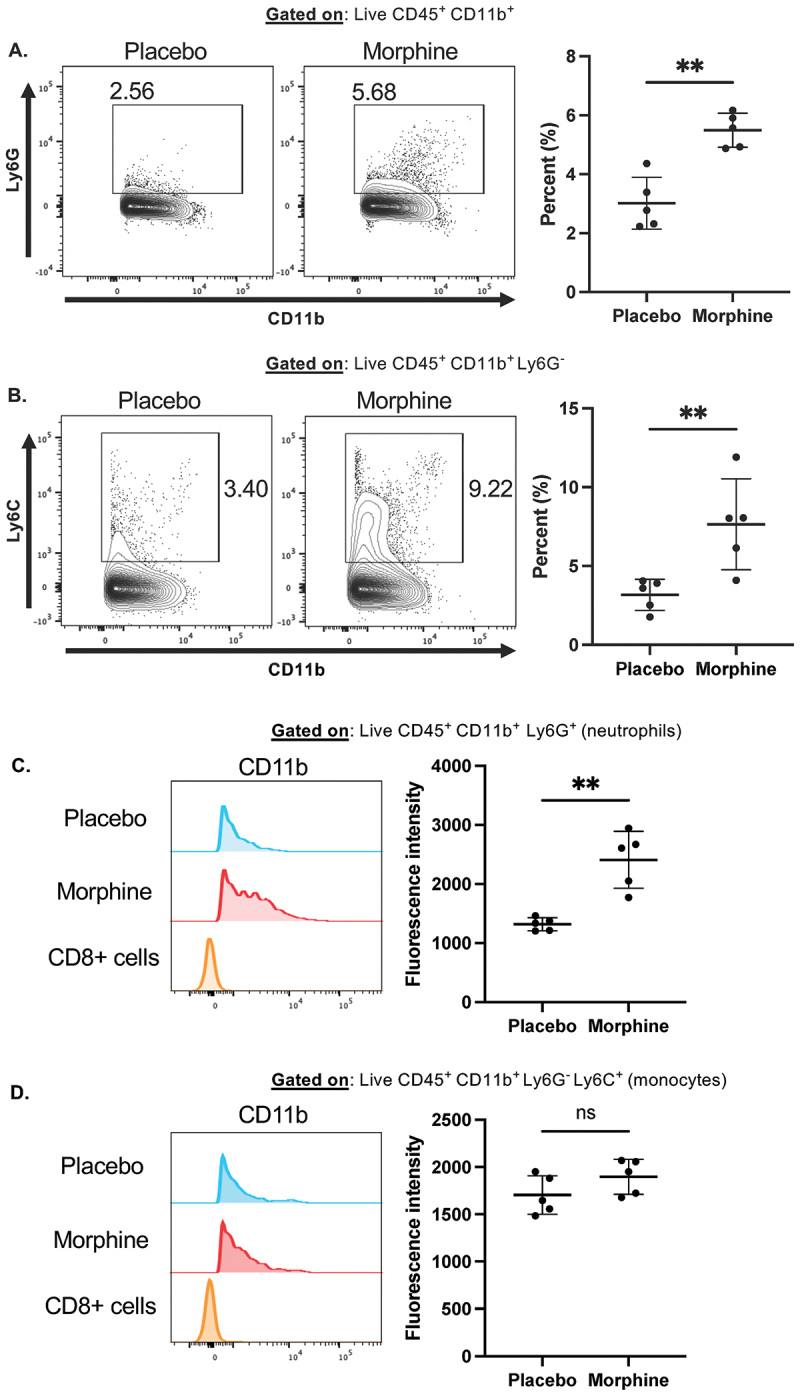
Intestine-infiltrating neutrophils increase CD11b expression during morphine treatment.

Myeloid cells conduct complement-mediated alterations to the microbiome. Particularly, CD11b^+^ neutrophils and monocytes are among the most important for complement-mediated phagocytosis of intestinal bacteria.^3^ We recently demonstrated that myeloid cells infiltrate the small intestine within 24 h of morphine treatment in a microbiome-dependent manner and play a significant role in morphine-induced microbial dysbiosis.^32^ Therefore, we postulated that intestinal myeloid cells may have increased expression of the complement receptor component CD11b during morphine treatment. First, we observed infiltration of Ly6G^+^ neutrophils and Ly6C^+^Ly6G^−^ monocytes at 24 h of morphine treatment (Figure 4A,B), as previously reported.^32^ We next determined the expression of CD11b on neutrophils and monocytes by mean fluorescence intensity (MFI) measurement. Intestine-infiltrating neutrophils, but not monocytes, exhibited increased MFI of CD11b during morphine treatment (Figure 4C,D). Increased myeloid cell expression of CD11b is associated with increased activation and effector function.^46,47^ These data are consistent with the previous report that intestinal complement-dependent bacterial clearance is mediated primarily by neutrophils.^3^ Together, this supports the notion that intestinal myeloid cells are poised to facilitate complement-dependent clearance of pathogenic bacteria during microbial dysbiosis.

(A-D). Flow cytometry on ileum lamina propria isolated 24 h after implantation with placebo or 25 mg morphine pellets (*n* = 5) (A). Representative flow cytometry plots and summary data of CD11b^+^Ly6G^+^ neutrophils (B). Representative flow cytometry plots and summary data of CD11b^+^ Ly6G^−^Ly6C^+^ monocytes (C). Representative histograms and summary data of CD11b expression on intestinal neutrophils (D). Representative histograms and summary data of CD11b expression on intestinal monocytes. Symbols represent individual mice. Mean and standard deviation are shown. Data points are pooled from two independent experiments. *p < 0.05 **p < 0.01 ***p < 0.001 ****p < 0.0001 using Mann Whitney U test (A–D).

C3 is emerging as an important regulator of intestinal homeostasis. The concentration of C3 correlates to the microbial composition of the intestine,^3^ C3-deficient mice experience constipation,^21^ and there is upregulation of intestinal complement genes during opioid treatment.^26,38^ Therefore, future studies using complement-deficient mice would further expand on the role of C3 in maintaining intestinal homeostasis between host and the microbiome.

Morphine induces microbial dysbiosis that causes expansion of pathogenic gram-positive bacteria and increased bacterial virulence.^29,48^ Expanding pathogenic gram-positive bacteria drive gut barrier disruption and subsequent bacterial translocation from the intestines into circulation.^24,25^ Bacterial translocation then results in a systemic immune response and inflammation.^49^ Here, we report rapid recruitment of the classical complement system to combat morphine-induced microbial dysbiosis.

**Figure 5.**
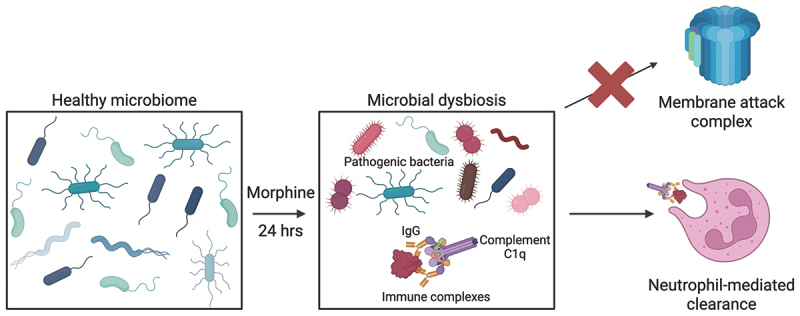
Schematic of proposed mechanism for intestinal recruitment and activation of the classical complement system during morphine-induced microbial dysbiosis.

This study shows that morphine induces a microbiome-dependent increase in the concentration of complement proteins in the ileal luminal content, with a corresponding increased concentration of antibodies. Further, increased immunoglobulins and complement proteins are accompanied by intestine-infiltrating myeloid cells with increased expression of CD11b on neutrophils. Taken together, this study demonstrates immediate involvement of the classical complement system during rapid morphine-induced microbial dysbiosis (Figure 5).

## Supplementary Material

Supplemental Material

5_1_25_GutMicrobesRep_Vitari_supplemental.docx

## Data Availability

The authors confirm that the data supporting the findings of this study are available within the article and its supplementary materials.
