# Supplementary material for: Gut microbial dysbiosis activates the classical complement pathway in a short-term morphine treatment model
Source: Gut Microbes Rep. 2025 Jul 13;2(1):2527628. doi: 10.1080/29933935.2025.2527628 (PMC12352340; doi:10.1080/29933935.2025.2527628)
Supplement: 5_1_25_GutMicrobesRep_Vitari_supplemental.docx [file KGMR_A_2527628_SM0738.docx]

**Figure S1***. The increased concentration of IgM and C1q in the ileal intestinal luminal content at 24 hrs of morphine treatment is not dependent on strain or sex.* Concentration of **A.** IgM and **B.** C1q determined by ELISA from the ileal luminal content of BALB/c mice 24 h after implantation with 25 mg morphine or placebo pellet (n = 10-11). Concentration of **C.** IgM and **D.** C1q determined by ELISA from the ileal luminal content of female B6 mice 24 h after implantation with 25 mg morphine or placebo pellet (n = 4-5). Symbols represent individual mice. Mean and SEM are shown. Data points are pooled from 2-3 independent experiments. * p < 0.05 ** p < 0.01 *** p < 0.001 **** p < 0.0001 using Mann Whitney U test **(A-D)**.

**Figure S2***. The concentrations of IgM and IgG3 are elevated in the ileal luminal content at 48hrs of morphine treatment.* Concentration of **A.** IgM and **B.** IgG3 determined by ELISA from the ileal luminal content of mice 48 h after implantation with 25 mg morphine or placebo pellet (n = 5-7). Symbols represent individual mice. Mean and SEM are shown. Data points are pooled from 2-3 independent experiments. * p < 0.05 ** p < 0.01 *** p < 0.001 **** p < 0.0001 using Mann Whitney U test **(A-B)**.

**Figure S3***. The concentration of IgM, but not IgG3, is elevated in the large intestinal luminal content at 24 and 48 hrs of morphine treatment.* Concentration of IgM and IgG3 determined by ELISA from the large intestinal luminal content of mice 24 h (**A** and **B**) or 48 hr (**C** and **D**) after implantation with 25 mg morphine or placebo pellet (n = 5-10). Symbols represent individual mice. Mean and SEM are shown. Data points are pooled from 2-3 independent experiments. * p < 0.05 ** p < 0.01 *** p < 0.001 **** p < 0.0001 using Mann Whitney U test **(A-D)**.
